# Prognostic value of CALLY index in patients with locally advanced non-small cell lung cancer treated with thoracic radiotherapy

**DOI:** 10.1186/s12885-026-16061-8

**Published:** 2026-04-24

**Authors:** Xiaoming Yin, Xinying He, Wei Guo, Hongling Lu, Haijun Chen, Rujing Huang, Tingting Hu, Li Xiao, Yunchuan Sun, Kui Fan

**Affiliations:** 1 Department of Radiation Oncology, Hebei Province Integrated Traditional Chinese and Western Medicine 3D Printing Technology Innovation Center, Hebei Province Cangzhou Hospital of Integrated Traditional and Western Medicine, Affiliated Hospital of Hebei Medical University, No. 31, Huanghe West Road, Yunhe District, Cangzhou, Hebei 061000 China; 2 Department of Anesthesiology, Cangzhou Central Hospital, Cangzhou, Hebei 061000 China

**Keywords:** C-reactive protein–albumin–lymphocyte (CALLY) index, Gross tumor volume (GTV), Locally advanced non-small cell lung cancer (LA-NSCLC), Radiotherapy(RT)

## Abstract

**Background:**

As a novel inflammatory-nutritional biomarker, the C-reactive protein–albumin–lymphocyte (CALLY) index has demonstrated significant prognostic value in several cancer types. However, its predictive significance for patients with locally advanced non-small cell lung cancer (LA-NSCLC) treated with thoracic radiotherapy (RT) remains elusive. This study aimed to investigate the relationship between the pre/post-treatment CALLY index and the prognosis of patients with LA-NSCLC treated with thoracic RT.

**Methods:**

In this retrospective study, a cohort of 218 LA-NSCLC patients who underwent thoracic RT between 2012 and 2018 was assessed. The calculation of CALLY involved the use of C-reactive protein (CRP) level, albumin level, and lymphocyte count. Cox proportional hazards regression and the Kaplan-Meier survival analyses were employed to analyze the relationships between various variables and overall survival (OS), local progression-free survival (LPFS), and distant metastasis-free survival (DMFS).

**Results:**

Multivariate Cox analysis revealed that post-treatment CALLY values were significantly associated with OS, LPFS, and DMFS. Patients with lower post-treatment CALLY values exhibited poorer survival outcomes compared with those with higher CALLY values.

**Conclusions:**

These findings demonstrate that the post-treatment CALLY index could be a valuable tool for predicting prognosis and guiding subsequent treatment strategies in patients with LA-NSCLC undergoing thoracic RT. Further prospective studies are warranted to validate these results and to explore the potential of incorporating the CALLY index into clinical decision-making.

**Supplementary Information:**

The online version contains supplementary material available at 10.1186/s12885-026-16061-8.

## Introduction

Although concurrent chemoradiotherapy (CCRT) followed by immune maintenance therapy has significantly enhanced the efficacy and is currently regarded as the standard treatment for inoperable locally advanced non-small cell lung cancer (LA-NSCLC), its 5-year survival rate is approximately 42.9% [1], highlighting the need for further improvements in treatment strategies. It is also noteworthy that in the real world, even with standard treatment methods, some patients still experience short-term recurrence and progression [2]. Additionally, some patients are unable to tolerate CCRT and receive only RT. Therefore, identifying eligible candidates for treatment and developing more targeted therapeutic strategies is crucial. In recent years, there has been growing attention to the role of the body’s inflammatory status, nutritional condition, and immune function in influencing tumor prognosis. Previous studies have demonstrated that inflammation plays an important role in various stages of tumor development, including onset, progression, malignant transformation, infiltration, and metastasis [3]. In addition, malignant tumors are associated with high metabolic demands, which often result in decreased appetite and impaired digestive and absorption functions in patients, leading to malnutrition. This condition may adversely influence prognosis [4]. Furthermore, impaired immune function, such as a decrease in lymphocytes, negatively impacts prognosis [5]. While RT and chemotherapy are effective in targeting tumors, they can induce several side effects, including loss of appetite, the release of pro-inflammatory factors, and a reduction in lymphocytes. Consequently, evaluating the impact of alterations in inflammatory status, nutritional condition, and immune function during RT on prognosis is of significant importance. Recently, a novel biomarker known as the CALLY index, which incorporates C-reactive protein (CRP), albumin, and lymphocyte levels, has been proposed. This index provides a comprehensive assessment of a patient’s inflammatory, nutritional, and immune status and has demonstrated substantial prognostic value in various malignancies [6–10]. However, there is currently no research investigating the relationship between CALLY index and prognosis of LA-NSCLC. As the pre-treatment CALLY represents the body’s baseline level, it has potential value for initial treatment strategies (such as regimen selection). Post-treatment CALLY reflects the body’s nutritional, immune, and inflammatory status after treatment and can guide subsequent management (such as follow-up intensity and adjuvant therapy). Therefore, the present study aimed to assess the predictive significance of CALLY index before and after treatment for the prognosis of LA-NSCLC, provide a reference for clinical physicians on the comprehensive management of them.

## Methods

### Patients

A total of 375 patients with unresectable LA-NSCLC were referred to our hospital for RT treatment between January 2012 and November 2018. Among them, 157 were excluded from the study due to missing key indicator data, including CRP levels for 59 patients, lymphocyte counts for 9 patients, and serum albumin levels for 89 patients. And the remaining 218 patients met the inclusion criteria after screening according to the following standards and were finally included in this study. The key inclusion criteria were as follows: age greater than 18 years; a diagnosis of NSCLC, including squamous cell carcinoma, adenocarcinoma, or large cell carcinoma; classification as stage IIIA, IIIB, or IIIC according to the AJCC 8th edition staging system; and completion of a standard course of fractionated RT. Participants were excluded if they presented with a secondary primary malignancy, pleural effusion, a history of prior thoracic surgery or RT, or any previous administration of targeted or immunotherapy.

The study protocol received approval from the Medical Ethics Committee of Cangzhou Hospital of Integrated Traditional Chinese and Western Medicine in Hebei Province (China; Approval No.2021-KY-062.1). All procedures were performed in compliance with pertinent guidelines and regulations. Written informed consent was obtained from each participant or legal guardian, with the forms emphasizing that consent was for research data/sample use only. Standard CCRT/RT was administered as part of clinical care, independent of enrollment in this study.

### Data collection and calculation of specific variables

All baseline patient information was obtained from the clinical medical record system, including age, sex, Eastern Cooperative Oncology Group (ECOG) score, smoking status, pathology, tumor (T) stage, lymph node (N) stage, TNM staging, the presence or absence of positron emission tomography/computed tomography (PET/CT) data, treatment methods (including whether CCRT was performed), and pre- and post-treatment levels of serum CRP, serum albumin, and lymphocyte count. Data were collected for all 218 patients both prior to RT and after the completion of the full RT regimen.

The pre/post-treatment CALLY was calculated using pre/post-treatment CRP level, serum albumin level, and the lymphocyte count. As previously described [10], CALLY index was calculated as follows: CALLY index = albumin (g/L) × lymphocyte count (10^9/L)/CRP (mg/L). Pre-treatment blood samples were collected at 6 a.m. the day before treatment, including patients receiving either RT alone or CCRT. Post-treatment blood samples were collected at 6 a.m. the day after the completion of treatment.

The RT method, radiation dosage and gross tumor volume (GTV) were extracted from the RT plan.

### Treatment delivery details

All patients undergo enhanced CT of the neck and chest for RT planning. Involved-field irradiation was utilized in all cases. Target volume delineation adhered to the definitions provided in ICRU Reports 62 and 83. Specifically, the Gross Tumor Volume (GTV) comprised disease extent identified via imaging and pathologic evaluation. The Clinical Target Volume (CTV) included regions of presumed microscopic involvement. The Planning Target Volume (PTV) was defined as the Internal Target Volume, incorporating margins for target motion, plus an additional setup margin to account for positioning and mechanical variability. Contouring of all organs at risk was conducted according to the Atlases for Organs at Risk in Thoracic RT. The total RT dose was 60–66 Gy / 30–33 fractions.

Patients received concurrent platinum-based chemotherapy. The specific chemotherapy regimen is as follows: carboplatin (AUC = 5, day1)/cisplatin (75 mg/m², day1) and etoposide (100 mg/m², day1-3), or carboplatin (AUC = 5, day1)/cisplatin (75 mg/m², day1) and pemetrexed (500 mg/m², day1), or carboplatin (AUC = 5, day1)/cisplatin (75 mg/m², day1) and paclitaxel (135-175 mg/m², day1) of each 21-day cycle, for a planned total of 2 cycles concurrently with RT.

### Statistical analysis

This study assessed three primary clinical endpoints, including overall survival (OS), locoregional progression-free survival (LPFS), and distant metastasis-free survival (DMFS). OS was measured from the date of initial diagnosis until death from any cause or the last follow-up. LPFS referred to the interval between diagnosis and the first occurrence of either locoregional disease progression or death. Similarly, DMFS was defined as the duration from diagnosis until the development of distant metastasis or death from any cause.

Continuous variables were presented as mean (Standard Deviation) when normally distributed or as median (interquartile range) for non-normally distributed variables. Categorical data were presented as percentage. Statistical differences between continuous or categorical variables were compared using Student’s t-test, Mann Whitney U test, Chi-square test, Fisher’s exact text, or logistic regression as appropriate. The non-parametric Wilcoxon signed-rank test was employed to compare paired samples with non-normal distributions. Both univariate and multivariate Cox proportional hazards regression analyses were conducted to assess the association between potential prognostic variables and outcomes. Key variables, such as pre/post-treatment CALLY and GTV volume were analyzed using Cox regression as continuous variables. Variables with *P*-value < 0.1 in the univariate analysis were subsequently included in the multivariate regression analysis. Based on the regression coefficients, hazard ratios (HRs) and their 95% confidence intervals (CI) were calculated. For visual descriptive purposes, patients were dichotomized based on the median of the post-treatment CALLY index to plot Kaplan-Meier survival curves. To describe the clinical characteristics of patients with different levels of the CALLY index in our cohort, the overall population was dichotomized into a high CALLY index group and a low CALLY index group based on the median value of the post-treatment CALLY index. Statistical analysis was carried out using SPSS 26.0 software (IBM, Armonk, NY, USA). *P* < 0.05 was considered statistically significant. To assess the validity of the proportional hazards assumption underlying the Cox regression model, the Schoenfeld residuals method was applied using SPSS 26.0 software. After executing the Cox regression, partial residuals were saved, the correlation between the residuals and the ranks of survival times was calculated, and Chi-square test was conducted for each covariate.

## Results

### Patient characteristics and treatment overview

A total of 218 patients were found eligible for inclusion in the analysis. The median follow-up time was 46.3 (42.9, 51.2) months, and 152 (69.7%) of patients died. 123 (80.9%) died due to lung cancer progression, and the remaining 29 patients (19.1%) died of unknown causes due to loss to follow-up or incomplete records. All patients were treated with three-dimensional conformal RT (3DRT) (4.1%) or intensity-modulated RT (IMRT) (79.4%) or volumetric modulated arc therapy (VMAT) (16.5%) to a median dose of 60 Gy (60–60). Among the population receiving CCRT, 80 (90.9%) patients underwent two cycles of concurrent chemotherapy, and 8 (9.1%) patients underwent one cycles of concurrent chemotherapy. The chemotherapy regimens were carboplatin/cisplatin and etoposide (67.9%), carboplatin/cisplatin and pemetrexed (27.5%), and carboplatin/-cisplatin and paclitaxel (4.6%). According to CTCAE v5.0, serious adverse events are defined as grade ≥ 4 adverse events. Among all patients in this study, 5 (2.3%) cases experienced grade IV neutropenia, and no other serious adverse events occurred. Characteristics of all patients, patients with CCRT and RT alone group was shown in Table S3.

### Association between variables and OS/LPFS/DMFS

**Fig. 1:**
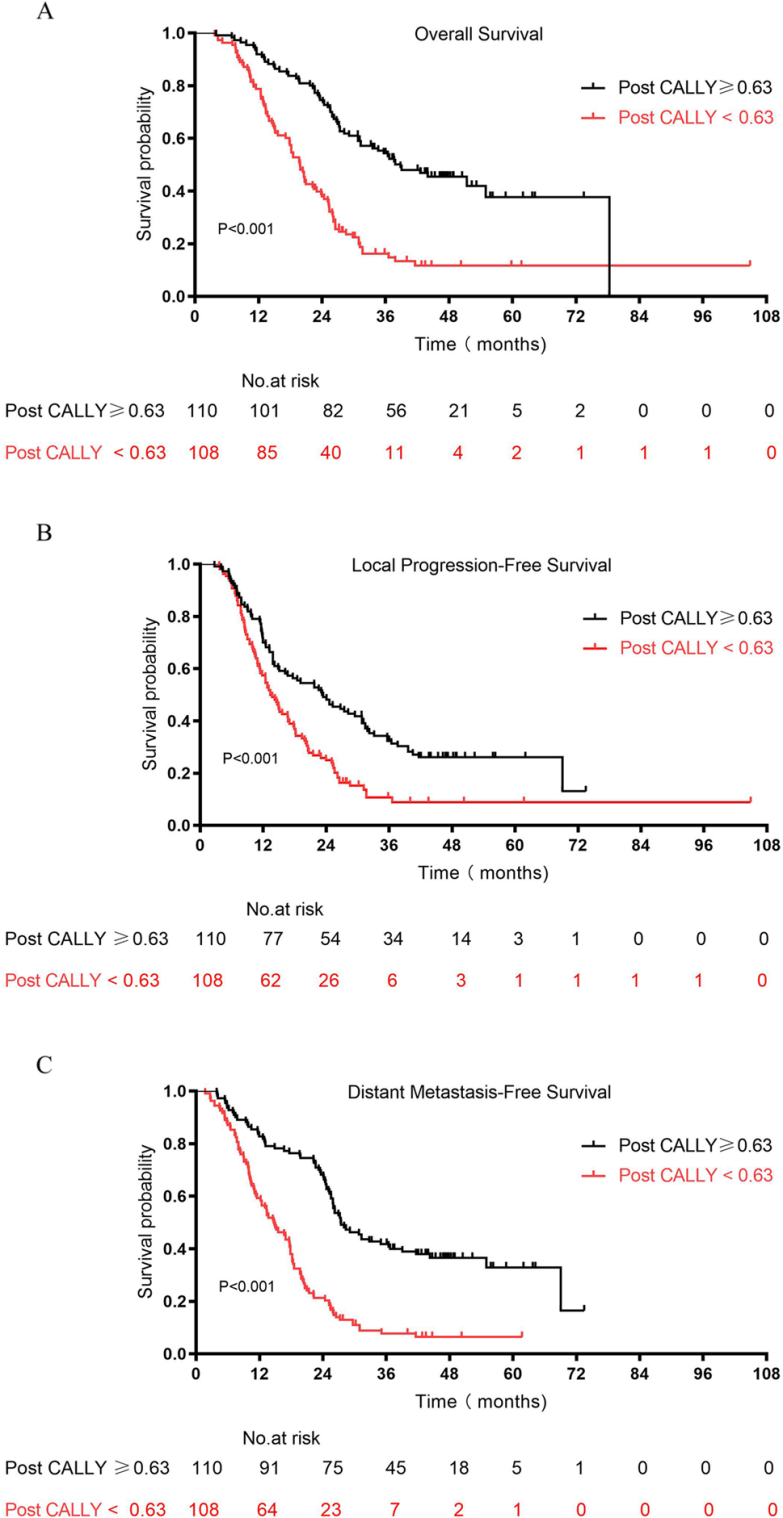
Kaplan–Meier curves for clinical outcomes by dichotomy (**A**) overall survival, (**B**) local progression-free survival, and (**C**) distant metastasis-free survival (DMFS). Patients were stratified by ranges of Post CALLY

The 5-year OS, LPFS, and DMFS rates were 25.4%, 11.8%, and 19.8%, with median values of 25.9, 16.7, and 20.5 months, respectively. On univariate analysis, T stage, CCRT, GTV, and pre/post-treatment CALLY showed a significant correlation with OS (*P* < 0.05). Smoking status, T stage, GTV, and pre/post-treatment CALLY exhibited a significant correlation with LPFS (*P* < 0.05). GTV and pre/post-treatment CALLY showed a significant correlation with DMFS (*P* < 0.05) (Table 1). On multivariate analysis, CCRT (*P* < 0.001), smaller GTV (*P* < 0.001), and higher post-treatment CALLY (*P* < 0.001) were associated with longer OS. Smaller GTV (*P* = 0.041) and higher post-treatment CALLY (*P* = 0.004) were associated with better LPFS. While post-treatment CALLY demonstrated a significant correlation with DMFS (*P* < 0.001) (Table 2). For visual descriptive purposes, post-treatment CALLY was dichotomized based on the median (0.63 (range, 0.43–1.06)) by Kaplan–Meier analysis for further analysis, in which lower post-treatment CALLY was associated with worse OS (*P* < 0.001) (Fig. 1A), poorer LPFS (*P* < 0.001) (Fig. 1B), and poorer DMFS (*P* < 0.001) (Fig. 1C).

**Table 1:** Univariable analysis of clinical and dosimetric variables with outcomes

Variables	OS		LPFS		DMFS	
	HR (95%CI)	P	HR (95%CI)	P	HR (95%CI)	P
Age (years)	1.003(0.984,1.022)	0.771	1.010(0.993,1.028)	0.249	1.003(0.985,1.020)	0.778
Sex	1.375(0.906,2.086)	0.135	1.462(1.996,2.146)	0.053	1.473(0.993,2.186)	0.054
ECOG-score	0.951(0.689,1.312)	0.759	0.947(0.703,1.276)	0.721	0.987(0.929,1.336)	0.930
Smoking history	1.519(0.994,2.320)	0.053	1.571(1.064,2.321)	0.023	1.159(0.789,1.702)	0.453
Histology		0.943		0.759		0.776
Tumor location	1.139(0.826,1.570)	0.429	1.033(0.766,1.394)	0.829	0.939(0.693,1.272)	0.685
T stage		0.040		0.013		0.143
N stage		0.252		0.235		0.165
Staging with PET	0.848(0.604,1.191)	0.342	1.218(0.890,1.668)	0.219	0.844(0.614,1.161)	0.294
Total RT dose	1.005(0.971,1.040)	0.789	1.009(0.977,1.043)	0.578	1.006(0.973,1.041)	0.711
Radiation technology		0.809		0.535		0.219
CCRT	0.697(0.499,0.974)	0.035	0.787(0.579,1.070)	0.126	0.845(0.618,1.153)	0.288
GTV (cm^3^)	1.006(1.004,1.008)	＜0.001	1.005(1.003,1.007)	<0.001	1.005(1.003,1.008)	＜0.001
Pre - CALLY	0.898(0.822,0.981)	0.017	0.922(0.853,0.966)	0.040	0.887(0.816,0.963)	0.004
Post - CALLY	0.241(0.149,0.389)	＜0.001	0.440(0.299,0.649)	＜0.001	0.219(0.140,0.345)	＜0.001

*Abbreviations: ECOG* Eastern Cooperative Oncology Group, *T* Tumor, *N* Node, *PET* Positron emission tomography, *RT* Radiation therapy, *CCRT* Concurrent Chemoradiotherapy, *GTV* Gross tumor volume, *Pre - CALLY* Pre-treatment C-reactive protein-albumin-lymphocyte, *Post-CALLY* Post-treatment C-reactive protein-albumin-lymphocyte, *HR* Hazard ratio, *OS* Overall survival, *LPFS* Local progression-free survival, *DMFS* Distant metastasis-free survival

**Table 2:** Multivariate analysis of clinical and dosimetric variables with outcomes

Variables	OS		LPFS		DMFS	
	HR (95%CI)	P	HR (95%CI)	P	HR (95%CI)	P
Sex			1.046(0.690,1.586)	0.832	1.043(0.691,1.574)	0.842
Smoking history	1.097(0.883,1.362)	0.402	1.394(0.926,2.099)	0.112		
T stage	1.096(0.924,1.229)	0.294	1.075(0.906,1.275)	0.406		
CCRT	0.541(0.378,0.775)	0.001				
GTV (cm^3^)	1.005(1.002,1.008)	0.001	1.003(1.000,1.006)	0.041	1.002(0.999,1.005)	0.123
Pre - CALLY	1.085(0.991,1.187)	0.077	1.027(0.950,1.111)	0.498	1.021(0.934,1.116)	0.645
Post - CALLY	0.269(0.156,0.464)	<0.001	0.521(0.333,0.816)	0.004	0.245(0.147,0.408)	<0.001

*Abbreviations: T* Tumor, *CCRT* Concurrent Chemoradiotherapy, *GTV* Gross tumor volume, *Pre - CALLY* Pre-treatment C-reactive protein-albumin-lymphocyte, *Post-CALLY* Post-treatment C-reactive protein-albumin-lymphocyte, *HR* Hazard ratio, *OS* Overall survival, *LPFS* Local progression-free survival, *DMFS* Distant metastasis-free survival

Because pre- and post-treatment parameters are strongly correlated, inclusion of both variables in the same multivariable Cox model may lead to overadjustment and multicollinearity. We calculated the correlation between the pre-treatment and post-treatment CALLY index (Pearson *r* = 0.488, *p* < 0.001), confirming that including both in the same model may be inappropriate. Therefore, we constructed models separately under the adjustment of the same covariates to fully evaluate the prognostic value of each parameter. Model 1 (pre-treatment CALLY only): there was no statistically significant difference between pre-treatment CALLY and OS (*P* = 0.764), LPFS (*P* = 0.531), and DMFS (*P* = 0.218) (Table S1) which similar with the above results. Model 2 (post-treatment CALLY only): there were also statistically significant differences between post-treatment CALLY and OS (*P* < 0.001), LPFS (*P* = 0.005), and DMFS (*P* < 0.001) ( Table S2).

While CCRT was found to improve OS, LPFS, and DMFS, its interaction with post-treatment CALLY values was not explored. This study further stratified post-treatment CALLY based on whether patients received CCRT or not. It was revealed that post-treatment CALLY was associated with OS in both the RT group [HR = 0.393, 95%CI 0.233–0.660, *p* < 0.001] and the CCRT group [HR = 0.043, 95%CI 0.014–0.136, *p* < 0.001]. Additionally, post-treatment CALLY was associated with LPFS in the RT group [HR = 0.581, 95%CI 0.372–0.908, *p* < 0.017] and in the CCRT group [HR = 0.215, 95%CI 0.101–0.458, *p* < 0.001]. Furthermore, it was related to DMFS in the RT group [HR = 0.343, 95%CI 0.205–0.572) *p* < 0.001] and in the CCRT group [HR = 0.069, 95%CI 0.027–0.177, *P* < 0.001]. Given the exceptionally low hazard ratio (HR = 0.043) for OS in the CCRT group, we accordingly present the corresponding Kaplan-Meier curves. These curves visually confirm that, when stratified by the median post-treatment CALLY, patients with high levels had significantly superior OS compared to those with low levels (*p* < 0.001) (Fig. S1).

### Characteristics of the population in high and low post-CALLY groups

The characteristics for all patients, as well as for those with high and low post-treatment CALLY indices, are presented in Table 3. Patients with a high CALLY index had a lower proportion of male gender compared with those with a low CALLY index. However, there was no significant difference in age, smoking status, ECOG-score, TNM stage, histology, PET staging, CCRT, RT technique, and location and delivered dose of RT between the two groups.

**Table 3:** Characteristics of all patients, patients with high and low Post-CALLY index

Characteristics	All patients (*n* = 218)	Patients with high Post-CALLY index (*n* = 110)	Patients with low Post-CALLY index (*n* = 108)	*P* value^d^
Sex^a^ (male)	175(80.3)	80(72.7)	95(88.0)	0.005
Age^b^ (year)	61(56,65)	61(57,66)	61(56,67)	0.594
Smoking status^a, c^ (Yes)	176(80.7)	85(77.3)	91(84.3)	0.191
ECOG-score^a^				0.054
0	121(55.5)	54(49.1)	67(62.0)	
1	97(44.5)	56(50.9)	41(38.0)	
T stage^a^				0.641
T1	31(14.2)	16(14.5)	15(13.9)	
T2	116(53.2)	62(56.4)	54(50.0)	
T3	27(12.4)	13(11.8)	14(13.0)	
T4	44(20.2)	19(17.3)	25(23.1)	
N stage^a^				0.310
N0	13(6.0)	6(5.5)	7(6.5)	
N1	12(5.5)	8(7.3)	4(3.7)	
N2	129(59.2)	69(62.7)	60(55.6)	
N3	64(29.4)	27(24.5)	37(34.2)	
TNM stage^a^				0.214
IIIA	114(52.3)	64(58.2)	50(46.3)	
IIIB	95(43.6)	42(38.2)	53(49.1)	
IIIC	9(4.1)	4(3.6)	5(4.6)	
Histology^a^				0.910
Squamous cell carcinoma	135(61.9)	68(61.8)	67(62)	
Adenocarcinoma	78(35.8)	39(35.5)	39(36.1)	
Large cell carcinoma	5(2.3)	3(2.7)	2(1.9)	
PET staging^a^ (Yes)	143(65.6)	69(62.7)	74(68.5)	0.368
CCRT^a^ (Yes)	88(40.4)	40(36.4)	48(44.4)	0.224
RT technique^a^				0.705
3D-CRT	9(4.1)	4(3.6)	5(4.6)	
IMRT	173(79.4)	90(81.8)	83(76.9)	
VMAT	36(16.5)	16(14.6)	20(18.5)	
Location^a^				0.265
Left lung	93(42.7)	51(46.4)	42(38.9)	
Right lung	125(57.3)	59(53.6)	66(61.1)	
Delivered dose of RT^b^ (Gy)	60(60,60)	60(60,60)	60(60,60)	0.677

*Abbreviations: Post-CALLY* Post-treatment C-reactive protein-albumin-lymphocyte, *ECOG* Eastern Cooperative Oncology Group, *T* Tumor, *N *Node, *M *Metastasis, *PET *Positron emission tomography, *CCRT* Concurrent Chemoradiotherapy, *RT* Radiation therapy, *3D-CRT* 3-dimensional conformal radiotherapy, *IMRT *Intensity modulated radiation therapy, *VMAT* Volumetric modulated arc therapy, *Gy* Gray

^a^Categorical variables are presented as number (percentage)

^b^Continuous variables are presented as median [interquartile range]

^c^The standard is to smoke more than 20 cigarettes in a lifetime

^d^The P value was for patients with high and low CALLY index

### Comparison of inflammatory makers before and after treatment between high and low post-CALLY groups

At baseline, significant differences were identified between the high and low post-treatment CALLY groups. Patients in the low post-treatment CALLY group presented with significantly higher levels of systemic inflammation, as evidenced by the elevated CRP level [31 (26, 34) mg/L vs. 23 (15, 29) mg/L, *P* < 0.001] and a lower lymphocyte count [1.59 (1.34, 1.95) ×10⁹/L vs. 1.92 (1.41, 2.39) ×10⁹/L, *P* < 0.001] compared with the high post-treatment CALLY group. No significant difference was identified in pre-treatment albumin levels between the two groups (*P* = 0.87).

Following treatment, the difference between the two groups further increased. The low post-treatment CALLY group demonstrated a significant exacerbation of systemic inflammation, with CRP level rising to 37 (30, 43) mg/L, which was significantly higher than the post-treatment CRP level in the high post-treatment CALLY group [28 (24, 32) mg/L, *P* < 0.001]. Conversely, both groups exhibited a decline in albumin level and lymphocyte count after therapy. However, this decline was significantly more severe in the low post-treatment CALLY group, resulting in significantly lower post-treatment albumin level [35 (34, 37) g/L vs. 37 (36, 39) g/L, *P* < 0.001] and lymphocyte count [0.43 (0.33, 0.52) ×10⁹/L vs. 0.81 (0.61, 1.00) ×10⁹/L, *P* < 0.001] compared with the high post-treatment CALLY group. Within-group analyses using the Wilcoxon signed-rank test confirmed that the changes from pre- to post-treatment were statistically significant for all three markers in both groups (all *P* < 0.001) (Table 4).

**Table 4:** Comparison of inflammatory makers before and after treatment between high and low Post-CALLY groups

Inflammatory maker	Time Point	high Post-CALLY index(*n* = 110)	low Post-CALLY index (*n* = 108)	*P* value (between groups)
CRP(mg/L)				
	Pre-treatment	23(15,29)	31(26,34)	< 0.001
	Post-treatment	28(24,32)	37(30,43)	< 0.001
	P value(within)	< 0.001	< 0.001	
serum albumin(g/L)				
	Pre-treatment	40(39,42)	40(39,42)	0.87
	Post-treatment	37(36,39)	35(34,37)	< 0.001
	P value(within)	< 0.001	< 0.001	
lymphocyte count(*10^9^/L)				
	Pre-treatment	1.92(1.41,2.39)	1.59(1.34,1.95)	< 0.001
	Post-treatment	0.81(0.61,1.00)	0.43(0.33,0.52)	< 0.001
	P value(within)	< 0.001	< 0.001	

*Abbreviations: Post-CALLY* Post-treatment C-reactive protein-albumin-lymphocyte, *CRP *C-reactive protein

### Data on serious adverse events

A statistical analysis on acute (reference standard: Common Terminology Criteria for Adverse Events, CTCAE 5.0) and late adverse (reference standard: Radiation Therapy Oncology Group, RTOG) events of radiation therapy based on CALLY values was conducted. We observed that the CALLY value is significantly associated with the occurrence of acute adverse events, particularly hematologic toxicities. Patients in the low CALLY group had a significantly higher incidence of Grade 3–4 acute leukopenia and neutropenia compared to the high CALLY group, both in the pre-CALLY and post-CALLY settings (all *P* < 0.05 ). Furthermore, for acute non-hematologic toxicities such as esophagitis and lung injury, the Low CALLY group also showed a significantly higher rate of severe (Grade 3–4) events in the post-CALLY phase ( *P* = 0.003 for esophagus; *P* = 0.016 for lung). However, regarding late adverse events (esophagus, heart, lung), our data did not reveal any statistically significant differences between the high and low CALLY groups (all *P* > 0.05 ) (Table S4).

## Discussion

In this study, a retrospective cohort was conducted specifically for LA-NSCLC patients to evaluate the prognostic value of the pre- and post-treatment CALLY index in outcomes following thoracic RT. The results demonstrated that post-treatment CALLY could serve as an independent prognostic factor for OS, LPFS, and DMFS.

Inflammation, nutrition, and immunity are critical factors in cancer progression [11–13]. Traditional indicators, such as the systemic immune-inflammation index (SII), neutrophil-to-lymphocyte ratio (NLR), platelet-to-lymphocyte ratio, lymphocyte-to-monocyte ratio, tumor-infiltrating lymphocytes (TILs), and the Prognostic Nutritional Index, have demonstrated certain predictive significance for tumor prognosis [14–16]. However, it is noteworthy that these indicators typically examine the impact of inflammation or nutritional status on tumor prognosis in isolation and rarely consider the combined effects of inflammation, nutrition, and immunity, in which the influences of all three factors on tumor prognosis cannot be disregarded. Recently, studies have highlighted the predictive value of the CALLY index in various cancer types, including lung cancer [6], breast cancer [7], esophageal cancer [8], colorectal cancer [9], and liver cancer [10]. The CALLY index, which incorporates CRP level, serum albumin level, and lymphocyte count, can reflect inflammation, nutritional status, and immune function. However, to date, there have been no reports on its prognostic value for unresectable LA-NSCLC. Therefore, this study explored the predictive value of pre-treatment CALLY for prognosis in patients with LA-NSCLC treated with RT. Equally important is that RT serves as a primary treatment method can also influence the body’s nutritional, inflammatory, and immune status. RT may lead to swallowing difficulties, weakening nutritional status [17], maintain an inflammatory environment through the release of damage-associated molecular patterns [18], and induce lymphopenia, negatively impacting prognosis [19]. Research has demonstrated that during RT for unresectable LA-NSCLC, the estimated dose of radiation to immune cells is an independent prognostic factor for distant metastasis-free survival [20], and changes in NLR before and after RT are independent prognostic factors for OS, LPFS, and DMFS [21]. These findings highlight the impact of RT on the body’s internal environment and its potential effect on prognosis. Therefore, the question arises: can the CALLY index after RT serve as an effective predictive factor for this patient population? The above issue is very important because pre-treatment parameters have potential value for initial treatment decisions (such as regimen selection); post-treatment parameters have unique significance for post-treatment risk re-stratification and subsequent management (such as follow-up intensity and adjuvant therapy).

Based on the above issues, this study explored the impact of pre/post-treatment CALLY and other clinical variables on the prognosis of RT in LA-NSCLC patients. It was found that pre-treatment CALLY could not be an independent prognostic factor for OS, LPFS, and DMFS, while post-treatment CALLY was noted as an independent prognostic factor for OS, LPFS, and DMFS. At the same time, the results of the independent multivariate Cox model were consistent with this which eliminated the influence of overadjustment and multicollinearity. For visual descriptive purposes, patients were dichotomized based on the median of the post-treatment CALLY index to plot Kaplan-Meier survival curves, and the results show that higher post-treatment CALLY values could be indicative of superior OS, LPFS, and DMFS. These results are not contradictory because pre-treament CALLY reflects baseline susceptibility, whereas post-treament CALLY is a composite indicator of baseline susceptibility and treatment tolerance/response, and therefore may contain more comprehensive prognostic information. These findings reflect that during treatment, interventions aimed at supporting nutrition and immune function may enhance overall treatment effectiveness for patients. To further explore the changes of inflammatory, nutritional, and immune status in the body before and after treatment, CRP level, albumin level, and lymphocyte count were compared according to the post-treatment CALLY index, revealing that patients with a low post-treatment CALLY index not only presented with a more remarkable pro-inflammatory and immunosuppressed state at diagnosis, but also experienced a significant exacerbation of these conditions following radical RT or CCRT. Specifically, the low post-treatment CALLY group exhibited escalating CRP level, and more severe reductions in albumin level and lymphocyte count, resulting in a significantly worse overall inflammatory-nutritional-immune status compared with the high post-treatment CALLY group. This finding is not unexpected, as RT itself can sustain an inflammatory environment by releasing damage-associated molecular patterns [18]. These results demonstrate that patients in poor physical condition may be more susceptible to the release of inflammatory factors, exacerbating malnutrition and reducing immune cell count. Albumin, a negative acute-phase protein, is synthesized at reduced levels in response to pro-inflammatory cytokines, such as IL-6, which are commonly elevated in a high-CRP state [22]. Hypoalbuminemia is associated with the reduced treatment tolerance, increased toxicity, and poorer overall survival, which aligns with the worse outcomes we observed in this group.

The abovementioned results suggest that the levels of inflammation, nutritional status, and immune function of the body after treatment may significantly impact prognosis.

Data on serious adverse events show the CALLY index is significantly correlated with acute adverse reactions to radiotherapy, especially hematological toxicity and some non hematological toxicity, but not significantly correlated with late adverse reactions. This result suggests that CALLY may reflect the patient’s short-term stress and tolerance to treatment, rather than a marker of long-term tissue repair or fibrosis risk. Notably, although univariate analysis revealed that pre-treatment CALLY had a statistically significant effect on OS, this effect was no longer statistically significant in the multivariate analysis. This further indicates that the overall condition of the body following treatment has a greater impact on prognosis, reflecting the role of clinicians in improving patient outcomes during treatment by enhancing nutrition, controlling inflammation, and supporting immune function. Furthermore, for patients with a low post-treatment CALLY value, more intensive treatments may be needed to further improve therapeutic efficacy. Similar to previous studies, this study indicated that CCRT is a favorable factor for improving OS [23, 24], and a larger GTV is associated with poorer OS and LPFS [25–27]. To explore whether CCRT modifies the relationship between post-treatment CALLY index and prognosis, the following stratified analysis was conducted: patients were divided into two groups based on whether they received CCRT, and Cox proportional hazards models were established to evaluate the association between CALLY index and survival outcomes. The results showed that post-treatment CALLY was associated with prognosis in both groups. It should be noted that in this study, the proportion of patients receiving concurrent CCRT was approximately 40.4%, a rate consistent with several real-world studies and reflective of actual clinical practice [28]. However, this figure appears lower than those reported in some large prospective clinical trials, which may be attributed to the complex interplay of multiple clinical factors-including performance status, comorbidities, and age-influencing treatment decisions in real-world settings. It is worth noting that although this study found that a larger GTV was associated with worse DMFS as evidenced by univariate analysis, this difference was not statistically significant in multivariate analysis. This reflects that GTV may be more strongly associated with local control, while systemic tumor control may be more closely related to the body’s levels of inflammation, nutritional status, and immune function.

However, the present study has several limitations. Firstly, its retrospective and single-center design might introduce selection bias and potential confounding variables, including the dosage of RT, the target area of RT, the method of RT-chemotherapy combination, and the chemotherapy regimen employed. Secondly, while the timing of post-treatment blood draws was standardized, it represents a singular time point and might not comprehensively reflect the dynamic recovery of inflammatory markers. Finally, the study did not analyze specific lymphocyte subsets, which could provide more valuable insights into the underlying immunological impairments.

## Conclusions

In this study, the post-treatment CALLY index emerged as a robust and biologically grounded prognostic marker for LA-NSCLC patients treated with RT. It encapsulates the deleterious interaction among treatment-aggravated systemic inflammation, nutritional decline, and severe treatment-induced lymphopenia. Early identification of this vulnerable phenotype using the post-treatment CALLY index provides a basis for personalized treatment strategies designed to interrupt this cycle, ultimately enhancing survival outcomes. Further studies are required to clarify the prognostic significance of post-treatment CALLY.

## Supplementary Information

Supplementary Material 1.

Supplementary Material 2.

Supplementary Material 3.

Supplementary Material 4.

Supplementary Material 5. Figure S1. Kaplan-Meier curves for overall survival by dichotomy in CCRT subgroup.

## Data Availability

The datasets generated during and/or analysed during the current study are available from the corresponding author on reasonable request.

## Abbreviations

CALLY: C-reactive protein-albumin-lymphocyte
LA-NSCLC: Locally advanced non-small cell lung cancer
RT: Radiation therapy
Pre-CALLY: Pre-treatment C-reactive protein-albumin-lymphocyte
Post-CALLY: Post-treatment C-reactive protein-albumin-lymphocyte
CRP: C-reactive protein
OS: Overall survival
LPFS: Local progression-free survival
DMFS: Distant metastasis-free survival
CCRT: Concurrent chemoradiotherapy
AJCC: American Joint Committee on Cancer
ECOG: Eastern Cooperative Oncology Group
T: Tumor
N: Node
PET: Positron emission tomography
CT: Computed tomography
GTV: Gross tumor volume
ICRU: International Commission on Radiation Units and Measurements
PTV: Planning Target Volume
AUC: Area Under the Curve
HR: Hazard ratio
CI: Confidence intervals
CTCAE: Common Terminology Criteria for Adverse Events
RTOG: Radiation Therapy Oncology Group
3D-CRT: 3-dimensional conformal radiotherapy
IMRT: Intensity modulated radiation therapy
VMAT: Volumetric modulated arc therapy
Gy: Gray
SII: Systemic immune-inflammation index
NLR: Neutrophil-to-lymphocyte ratio
TILs: Tumor-infiltrating lymphocytes
