# Supplementary material for: Prognostic value of CALLY index in patients with locally advanced non-small cell lung cancer treated with thoracic radiotherapy
Source: BMC Cancer. 2026 Apr 24;26:722. doi: 10.1186/s12885-026-16061-8 (PMC13244888; doi:10.1186/s12885-026-16061-8)
Supplement: Supplementary file 1 — Supplementary Material 1. [file 12885_2026_16061_MOESM1_ESM.docx]

**Table S1** Multivariate analysis of clinical and dosimetric variables with outcomes（Model 1）

OS LPFS DMFS

Variables

HR（95%CI） P HR（95%CI） P HR（95%CI） P

Sex 1.039(0.842,1.281) 0.723 1.094(0.892,1.343) 0.389

Smoking history 1.136(0.857,2.022) 0.210 1.433(0.950,2.163) 0.086

T stage 1.094(0.922,1.299) 0.301 1.070(0.903,1.267) 0.436

CCRT 0.564(0.392,0.809) 0.002

GTV (cm^3^) 1.007(1.004,1.009) <0.001 1.004(1.001,1.006) 0.005 1.004(1.002,1.007) 0.001

Pre - CALLY 1.316(0.857,2.022) 0.764 0.978(0.912,1.049) 0.531 0.949(0.873,1.031) 0.218

*Abbreviations:* T = tumor; CCRT = Concurrent Chemoradiotherapy; GTV= gross tumor volume; Pre - CALLY = Pre-treatment C-reactive protein-albumin-lymphocyte; HR = hazard ratio; OS = overall survival; LPFS = local progression-free survival; DMFS = distant metastasis-free survival.
