# Supplementary material for: Prognostic value of CALLY index in patients with locally advanced non-small cell lung cancer treated with thoracic radiotherapy
Source: BMC Cancer. 2026 Apr 24;26:722. doi: 10.1186/s12885-026-16061-8 (PMC13244888; doi:10.1186/s12885-026-16061-8)
Supplement: Supplementary file 2 — Supplementary Material 2. [file 12885_2026_16061_MOESM2_ESM.docx]

**Table S2** Multivariate analysis of clinical and dosimetric variables with outcomes（Model 2）

OS LPFS DMFS

Variables

HR（95%CI） P HR（95%CI） P HR（95%CI） P

Sex 1.021(0.829,1.258) 0.844 1.021(0.831,1.255) 0.842

Smoking history 1.207(0.783,1.861) 0.395 1.397(0.928,2.103) 0.109

T stage 1.104(0.930,1.310) 0.257 1.076(0.907,1.276) 0.401

CCRT 0.573(0.403,0.815) 0.002

GTV (cm^3^) 1.004(1.001,1.007) 0.006 1.003(1.000,1.005) 0.054 1.002(0.999,1.005) 0.143

Post - CALLY 0.316(0.190,0.525) <0.001 0.554(0.368,0.833) 0.005 0.253(0.155,0.413) <0.001

*Abbreviations:* T = tumor; CCRT = Concurrent Chemoradiotherapy; GTV= gross tumor volume; Post-CALLY = Post-treatment C-reactive protein-albumin-lymphocyte; HR = hazard ratio; OS = overall survival; LPFS = local progression-free survival; DMFS = distant metastasis-free survival.
