# Supplementary material for: Prognostic value of CALLY index in patients with locally advanced non-small cell lung cancer treated with thoracic radiotherapy
Source: BMC Cancer. 2026 Apr 24;26:722. doi: 10.1186/s12885-026-16061-8 (PMC13244888; doi:10.1186/s12885-026-16061-8)
Supplement: Supplementary file 3 — Supplementary Material 3. [file 12885_2026_16061_MOESM3_ESM.docx]

**Table S3** Characteristics of all patients, patients with CCRT and RT alone group.

| **Characteristics** | **All patients(n = 218)** | **CCRT (n = 88)** | **RT alone(n = 130)** | **P value^c^** |
| --- | --- | --- | --- | --- |
| Age,years |  |  |  |  |
| Age≥61^a^,n(%) | 113(51.8) | 41(46.6) | 72(55.4) | 0.203 |
| Median(IQR)^b^ | 61(56-65) | 60(57-66) | 62(57-69) | 0.030 |
| Comorbidities present^a^,n(%) | 54(24.8) | 15(17.0) | 39(30.0) | 0.030 |
| ECOG-score^a^ |  |  |  |  |
| 0 | 121(55.5) | 52(59.1) | 69(53.1) | 0.381 |
| 1 | 97(44.5) | 36(40.9) | 61(46.9) | 0.381 |

Abbreviations: CCRT = Concurrent Chemoradiotherapy; RT = radiation therapy; ECOG = Eastern Cooperative Oncology Group.

Comorbidities: including hypertension, coronary heart disease, and diabetes.

^a^Categorical variables are presented as number (percentage).

^b^Continuous variables are presented as median [interquartile range].

^c^The P value was for patients with CCRT and RT alone group.
