# Supplementary material for: Prognostic value of CALLY index in patients with locally advanced non-small cell lung cancer treated with thoracic radiotherapy
Source: BMC Cancer. 2026 Apr 24;26:722. doi: 10.1186/s12885-026-16061-8 (PMC13244888; doi:10.1186/s12885-026-16061-8)
Supplement: Supplementary file 4 — Supplementary Material 4. [file 12885_2026_16061_MOESM4_ESM.docx]

**Table S4** Acute（CTCAE 5.0）and late (RTOG) adverse events of RT with high and low pre/post CALLY

| **Variable** |  |  | Pre-CALLY | | P | Post-CALLY | | p |
| --- | --- | --- | --- | --- | --- | --- | --- | --- |
|  |  |  | High(n=110) | Low(n=108) |  | High(n=110) | Low(n=108) |  |
| Acute adverse events of RT | Grade |  |  |  |  |  |  |  |
| White blood cell |  |  |  |  |  |  |  |  |
|  | 0-2 |  | 106(96.4) | 86(79.4) | <0.001 | 105(95.5) | 87(80.6) | 0.001 |
|  | 3-4 |  | 4(3.6) | 22(20.4) |  | 5(4.5) | 21(19.4) |  |
| Neutrophils |  |  |  |  |  |  |  |  |
|  | 0-2 |  | 106(96.4) | 86(79.6) | <0.001 | 105(95.5) | 87(80.6) | 0.001 |
|  | 3-4 |  | 4(3.6) | 22(20.4) |  | 5(4.5) | 21(19.4) |  |
| Hemoglobin |  |  |  |  |  |  |  |  |
|  | 0-2 |  | 106(96.4) | 103(95.4) | 0.978 | 107(97.3) | 102(94.4) | 0.478 |
|  | 3-4 |  | 4(3.6) | 5(4.6) |  | 3(2.7) | 6(5.6) |  |
| Platelet |  |  |  |  |  |  |  |  |
|  | 0-2 |  | 107(97.3) | 101(93.5) | 0.317 | 107(97.3) | 101(93.5) | 0.317 |
|  | 3-4 |  | 3(2.7) | 7(6.5) |  | 3(2.7) | 7(6.5) |  |
| Esophagus |  |  |  |  |  |  |  |  |
|  | 0-2 |  | 107(97.3) | 96(88.9) | 0.014 | 108(98.2) | 95(88.0) | 0.003 |
|  | 3-4 |  | 3(2.7) | 12(11.1) |  | 2(1.8) | 13(12.0) |  |
| Heart |  |  |  |  |  |  |  |  |
|  | 0-2 |  | 110(100) | 108(100) | - | 110(100) | 108(100) | - |
|  | 3-4 |  | 0(0.0) | 0(0.0) |  | 0(0.0) | 0(0.0) |  |
| Lung |  |  |  |  |  |  |  |  |
|  | 0-2 |  | 106(96.4) | 100(92.6) | 0.222 | 108(98.2) | 98(90.7) | 0.016 |
|  | 3-4 |  | 4(3.6) | 8(7.4) |  | 2(1.8) | 10(9.3) |  |
| Late adverse events of RT |  |  |  |  |  |  |  |  |
| Esophagus |  |  |  |  |  |  |  |  |
|  | 0-2 |  | 106(96.4) | 102(94.4) | 0.724 | 107(97.3) | 101(93.5) | 0.317 |
|  | 3-4 |  | 4(3.6) | 6(5.6) |  | 3(2.7) | 7(6.5) |  |
| Heart |  |  |  |  |  |  |  |  |
|  | 0-2 |  | 110(100) | 108(100) | - | 110(100) | 108(100) | - |
|  | 3-4 |  | 0(0.0) | 0(0.0) |  | 0(0.0) | 0(0.0) |  |
| Lung |  |  |  |  |  |  |  |  |
|  | 0-2 |  | 107(97.3) | 105(97.2) | 1.000 | 108(98.2) | 104(96.3) | 0.662 |
|  | 3-4 |  | 3(2.7) | 3(2.8) |  | 2(1.8) | 4(3.7) |  |

Abbreviations: CTCAE:Common Terminology Criteria for Adverse Events; RTOG:Radiation Therapy Oncology Group; RT = radiation therapy; Pre-CALLY = Pre-treatment C-reactive protein-albumin-lymphocyte; Post-CALLY = Post-treatment C-reactive protein-albumin-lymphocyte
