# Supplementary figures and images for: Prognostic value of CALLY index in patients with locally advanced non-small cell lung cancer treated with thoracic radiotherapy
Source: BMC Cancer. 2026 Apr 24;26:722. doi: 10.1186/s12885-026-16061-8 (PMC13244888; doi:10.1186/s12885-026-16061-8)

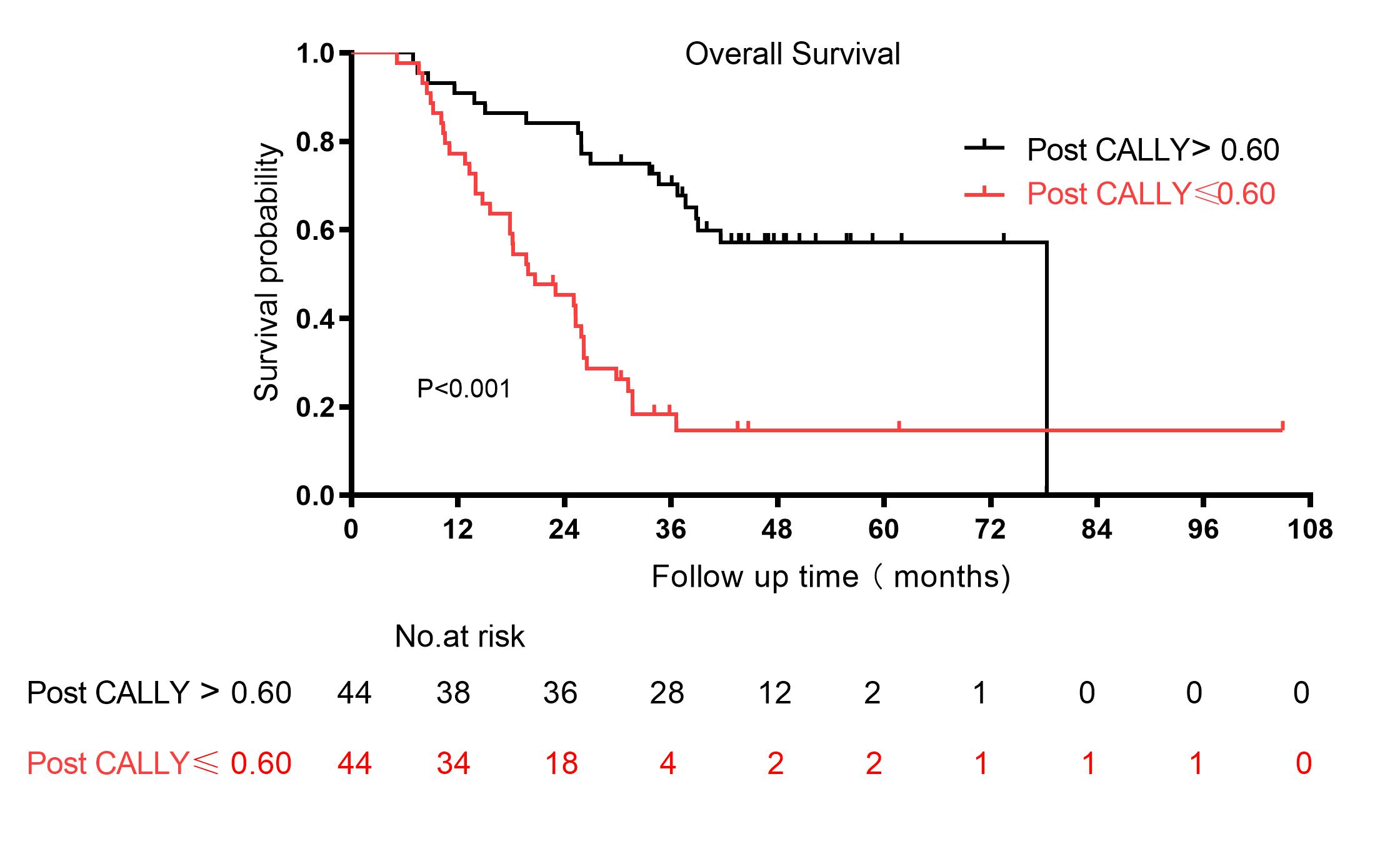

Supplement: Supplementary file 5 — Supplementary Material 5. Figure S1. Kaplan-Meier curves for overall survival by dichotomy in CCRT subgroup. [file 12885_2026_16061_MOESM5_ESM.jpg]
